# Supplementary material for: Structural basis of R-loop recognition by the S9.6 monoclonal antibody
Source: Nat Commun. 2022 Mar 28;13:1641. doi: 10.1038/s41467-022-29187-7 (PMC8960830; doi:10.1038/s41467-022-29187-7)
Supplement: Supplementary file 1 — Supplementary Information [file 41467_2022_29187_MOESM1_ESM.pdf]

Supplementary Materials for:

Structural basis of R-loop recognition by the S9.6 monoclonal antibody

Charles Bou-Nader, Ankur Bothra, David N. Garboczi, Stephen H. Leppla, and Jinwei Zhang

Supplementary Tables 1-2
Supplementary Figures 1-9

Nucleic Acid	% GC content	S9.6 Fab	K_d (μM)	Log(K_a)
13-bp dsDNA		WT	N.D.	
13-bp dsRNA			3.79 ± 0.41	5.42 ± 0.05
		HC_Y54F	8.3 ± 0.8	5.08 ± 0.04
		HC_Y54W	11.8 ± 2.3	4.93 ± 0.08
		HC_Y54H	4.3 ± 0.4	5.37 ± 0.03
		HC_Y54R	1.91 ± 0.3	5.72 ± 0.06
		HC_Y54Q	1.64 ± 0.2	5.79 ± 0.05
		HC_Y54A	2.35 ± 0.35	5.63 ± 0.06
		HC_Y54L	3.99 ± 1.1	5.4 ± 0.11
		HC_Y54V	1.61 ± 0.25	5.79 ± 0.07
		HC_Y54M	4.53 ± 2.1	5.35 ± 0.11
13-bp DNA-RNA hybrid		WT	0.19 ± 0.02	6.72 ± 0.06
		HC_S31A	0.14 ± 0.02	6.85 ± 0.06
		HC_S31G	0.17 ± 0.01	6.77 ± 0.04
		HC_S31L	N.D.	
		HC_Y32A	25.4 ± 17	4.72 ± 0.05
		HC_Y54F	0.4 ± 0.01	6.39 ± 0.01
		HC_Y54W	0.76 ± 0.46	6.17 ± 0.27
		HC_Y54H	0.36 ± 0.08	6.45 ± 0.1
		HC_Y54R	0.15 ± 0.01	6.82 ± 0.04
		HC_Y54Q	0.73 ± 0.14	6.14 ± 0.08
		HC_Y54D	5.16 ± 2.3	5.29 ± 0.19
		HC_Y54A	2.65 ± 0.5	5.58 ± 0.09
		HC_Y54L	1.93 ± 0.6	5.71 ± 0.14
		HC_Y54V	1.24 ± 0.1	5.91 ± 0.05
		HC_Y54M	1.41 ± 0.1	5.85 ± 0.05
		HC_Y54G	11.35 ± 3.7	4.96 ± 0.14
		HC_N55A	0.27 ± 0.11	6.58 ± 0.18
		HC_N55R	0.42 ± 0.12	6.39 ± 0.13
		HC_Y100A	0.13 ± 0.01	6.89 ± 0.03
		HC_Y100F	0.17 ± 0.03	6.79 ± 0.08
		HC_Y101A	N.D.	
		HC_Y101F	0.32 ± 0.07	6.5 ± 0.09
		HC_G102A	5.3 ± 1.37	5.3 ±0.13
		HC_G102L	N.D.	
		HC_S103A	0.22 ± 0.01	6.66 ± 0.03
		HC_S103L	0.13 ± 0.01	6.89 ± 0.04
		HC_R104A	N.D.	
		LC_N33A	1.16 ± 0.24	5.94 ± 0.09
		LC_N33K	0.22 ± 0.03	6.67 ± 0.07
		LC_Y54A	0.12 ± 0.04	6.94 ± 0.16
		LC_Y54F	0.16 ± 0.08	6.83 ± 0.24
		LC_H31A	1.91 ± 0.4	5.73 ± 0.09
		LC_Y37F	0.37 ± 0.03	6.44 ± 0.04
		LC_C219A	0.11 ± 0.002	6.95 ± 0.01
dG4C:rC10G, 13-bp hybrid		WT	0.18 ± 0.03	6.75 ± 0.08
rU7A:dA7T, 13-bp hybrid			0.11 ± 0.02	6.95 ± 0.07

2′-OH (Uridine) at dT5, 13-bp hybrid			0.14 ± 0.01	6.87 ± 0.04
2′-OH at dT3-dG4-dT5-dC6-dA7, in 13-bp hybrid			0.1 ± 0.01	7.0 ± 0.05
2′-OH at U7-G8-A9, in 13-bp dsDNA			7.0 ± 1.9	5.15 ± 0.11
dT3-dC6-dA9-dC12 in RNA strand, 13-bp hybrid			0.38 ± 0.05	6.4 ± 0.06
dT2-dG4-dC6-dG8-dC10-dC12 in RNA strand, 13-bp hybrid			20.6 ± 5.8	4.68 ± 0.12
10-bp DNA-RNA hybrid	0		N.D.	
	10		N.D.	
	20		N.D.	
	30		N.D.	
	40		N.D.	
	50		3.11 ± 0.2	5.5 ± 0.1
	60		0.61 ± 0.08	6.21 ± 0.01
	70		0.42 ± 0.03	6.37 ± 0.04
	80		0.57 ± 0.08	6.24 ± 0.03
	90		1.02 ± 0.14	6.01 ± 0.06
	100		2.61 ± 0.9	5.55 ± 0.12
15-bp DNA-RNA hybrid	0		N.D.	
13-bp dsDNA from human *FUS* locus			N.D.	
13-bp dsRNA from human *FUS* locus			5.36 ± 0.9	5.27 ± 0.07
13-bp hybrid from human *FUS* locus			0.08 ± 0.01	7.11 ± 0.08
13-bp dsDNA from β-actin terminator region			N.D.	
13-bp dsRNA from β-actin terminator region			1.52 ± 0.26	5.82 ± 0.07
13-bp hybrid from β-actin terminator region			0.09 ± 0.01	7.03 ± 0.08

Supplementary Table 1. Summary of fluorescence polarization titrations of nucleic acids with S9.6.

HC: heavy chain; LC: light chain. N.D.: not determined due to weak binding. Values are mean ± s.d. n= 3 biologically independent samples. Source data are provided as a Source Data file.

	Free S9.6 Fab<br>PDB: 7TQA	S9.6 Fab bound to a<br>13-bp DNA-RNA hybrid<br>PDB: 7TQB
**Data collection**		
Space group	P 31	P 1 21 1
Cell dimensions		
a, *b*, *c* (Å)	96.65, 96.65, 140.58	38.43, 158.88, 43.14
α, β, γ (°)	90, 90, 120	90, 105.15, 90
Resolution (Å)	45.7 – 2.33 (2.41 – 2.33)	36.12 – 3.1 (3.211 – 3.1)
R_{sym} or R_{merge}	0.2301 (2.709)	0.2425 (0.533)
$I / \sigma I$	9.24 (1.55)	2.12 (1.16)
$CC_{1/2}$	0.996 (0.522)	0.835 (0.779)
CC*	0.999 (0.828	0.944 (0.936)
Completeness (%)	99.57 (99.35)	95.89 (90.89)
Redundancy	14.6 (12.5)	2.7 (2.5)
**Refinement**		
Resolution (Å)	45.7 – 2.33 (2.41 – 2.33)	36.12 – 3.1 (3.211 – 3.1)
No. reflections	62725 (6284)	8682 (818)
R_{work} / R_{free}	0.1812/0.2352<br>(0.2631/0.3369)	0.2295/0.2724<br>(0.2743/0.2752)
No. atoms		
Macromolecule	9846	3799
Water	418	36
Ligand	66	6
No. nucleotides		
DNA	-	13
RNA	-	13
R.M.S. deviations		
Bond lengths (Å)	0.008	0.004
Bond angles (°)	1.02	0.78
Ramachandran plot (%)		
Favored	94.7	94.7
Allowed	5.3	5.3
Disallowed	0.0	0.0

Supplementary Table 2. Summary of data collection and refinement statistics for the crystal structures of free S9.6 Fab and S9.6 Fab bound to a 13-bp DNA-RNA hybrid duplex.

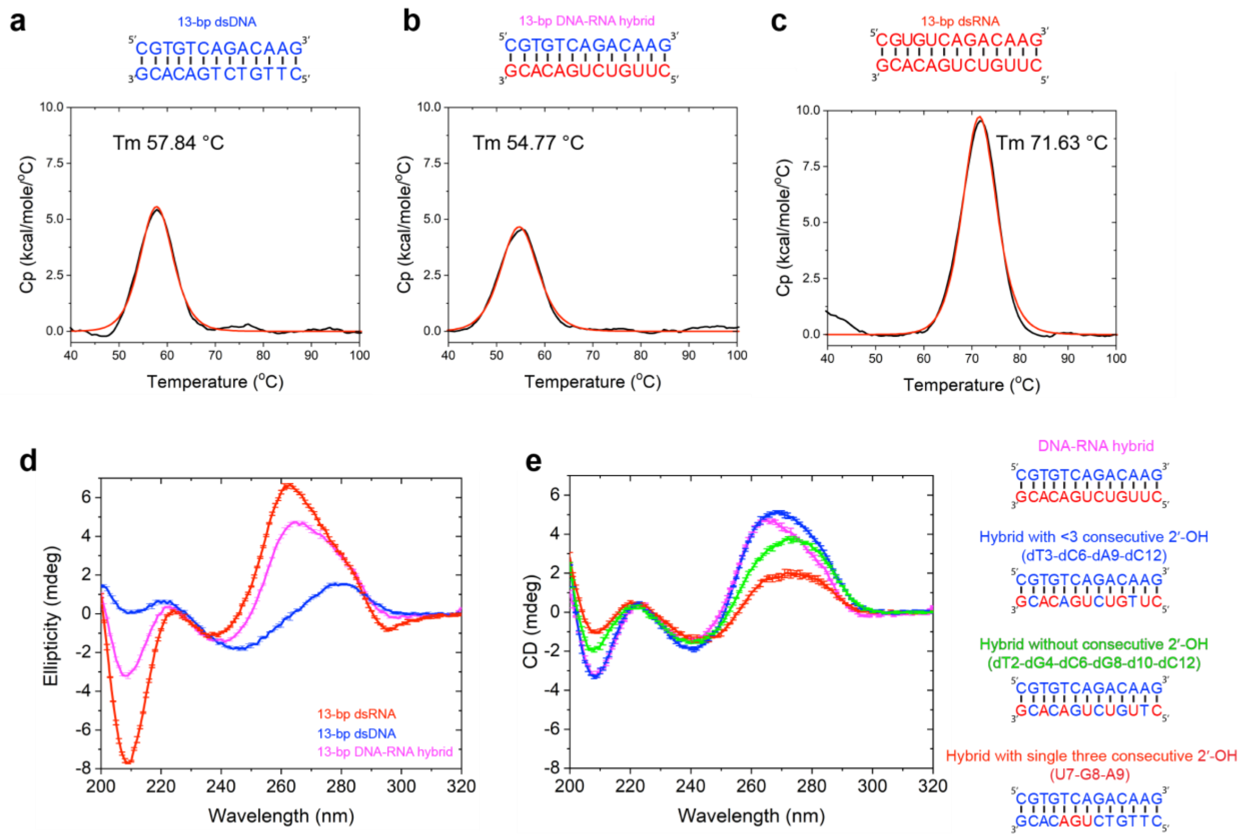

Supplementary Figure 1. Biophysical characterization of the stability of 13-bp duplexes. (a-c) Differential scanning calorimetry (DSC) analysis of 13-bp duplexes. Sequences of double-stranded (ds) nucleic acids used are shown on top of each panel with RNA and DNA indicated in red and blue, respectively. The measured melting temperature (T_m) is indicated. **d** Circular dichroism (CD) measurement of the 13-bp duplexes shown in **(a-c)**. **e** CD measurement of hybrids containing varying numbers of ribonucleotides with their sequences shown on the right. All CD values are mean ± s.d. n= 3 biologically independent samples. Source data are provided as a Source Data file.

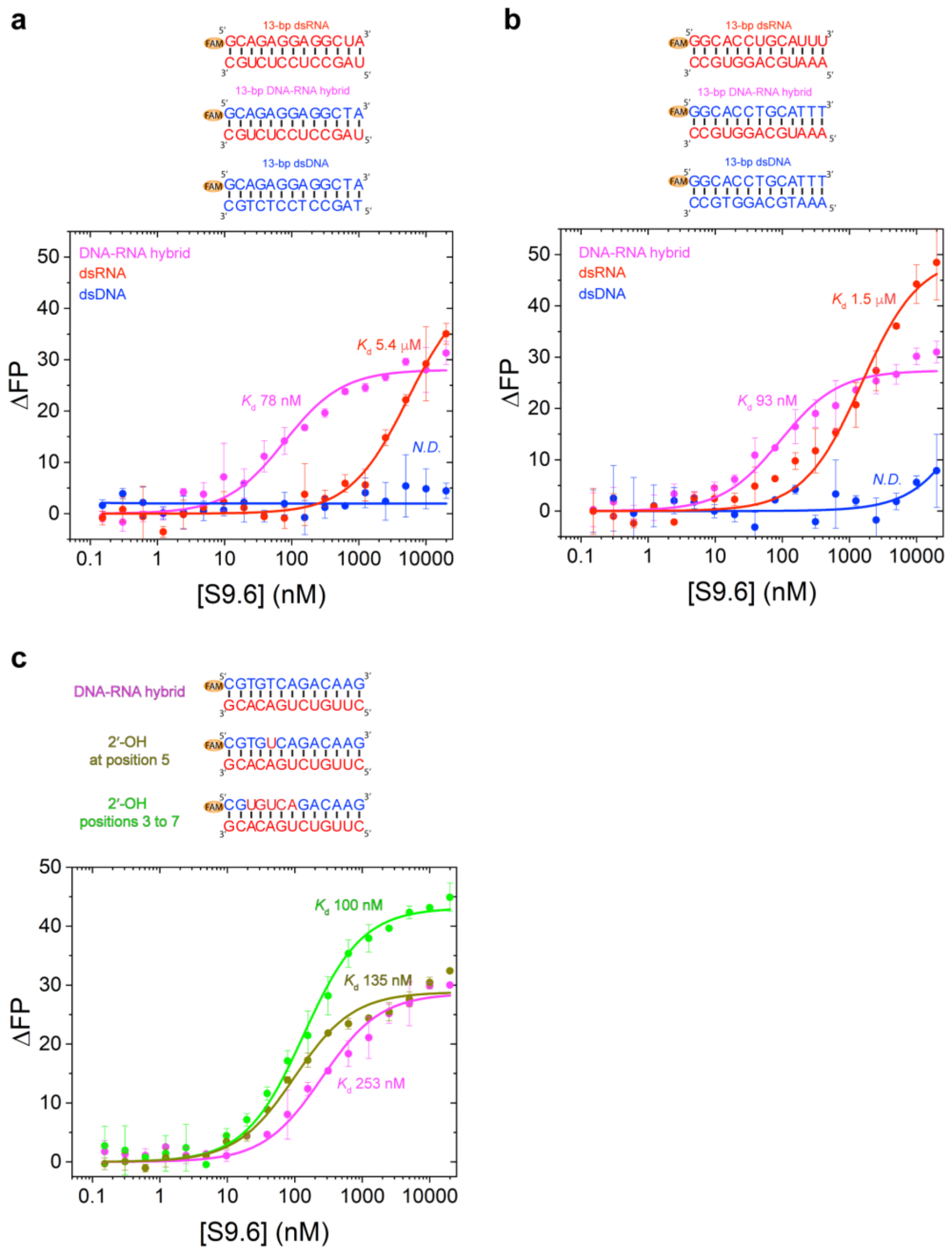

Supplementary Figure 2. Biophysical characterizations of nucleic acid binding preferences of S9.6 Fab. a-b Fluorescence polarization titrations of S9.6 with different types of nucleic acids derived from 13-bp segments of the R-loops from the human *FUS* locus (**a**)[1] and the β-actin terminator region (**b**)[2]. **c** Fluorescence polarization titrations of S9.6 with different DNA-RNA hybrids that contain ribonucleotides in the DNA strand. Sequences of double-stranded (ds) nucleic acids used for S9.6 characterizations and locations of FAM labels are shown on top of each panel with RNA and DNA nucleotides indicated in red and blue, respectively. Apparent binding constants (K_ds) are indicated. ΔFP: changes in fluorescence polarization, in mP units. N.D.: not determined. Values are mean ± s.d. n= 3 biologically independent samples. Source data are provided as a Source Data file.

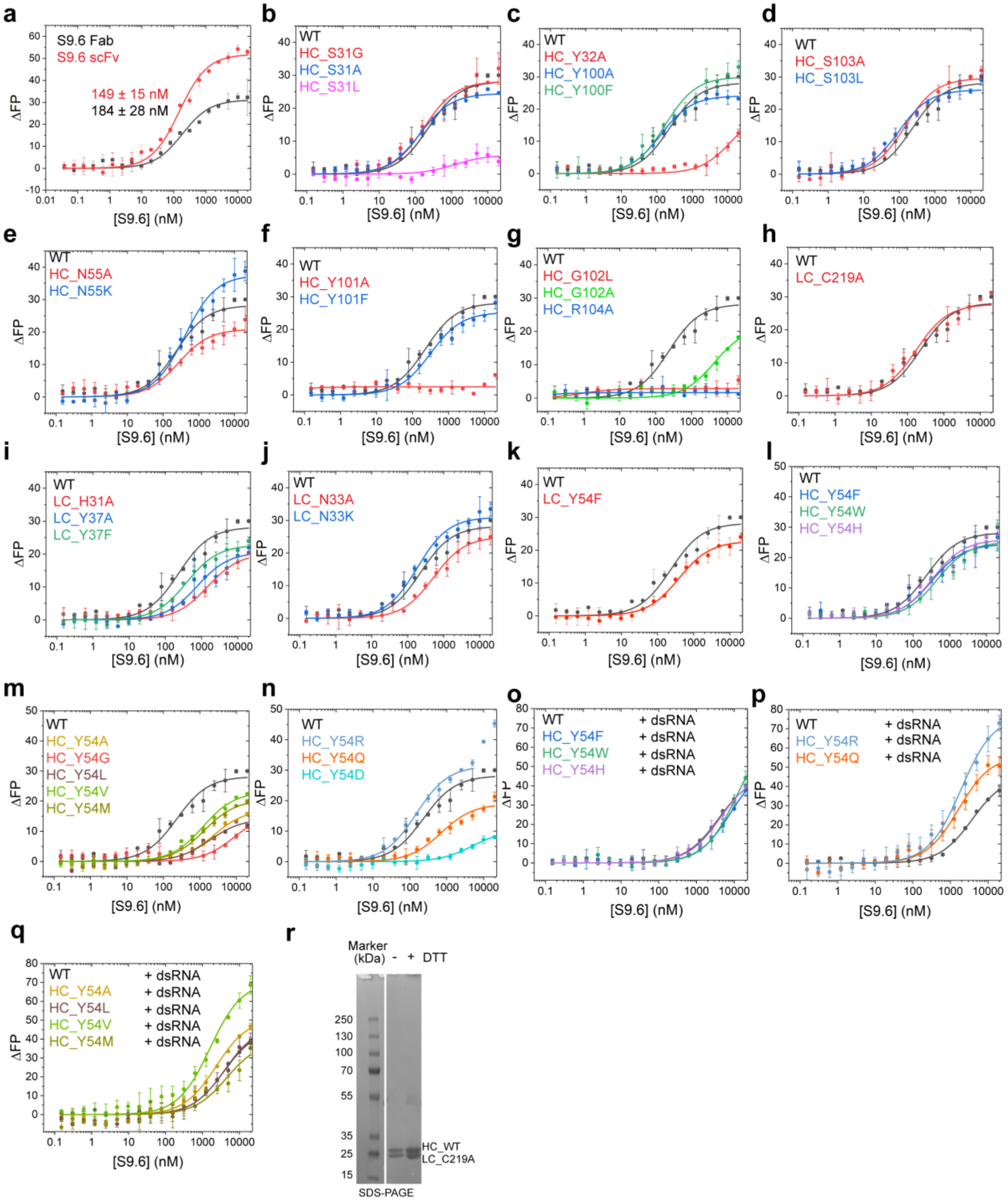

a
S9.6 Fab
S9.6 scFv
149 ± 15 nM
184 ± 28 nM
ΔFP
[S9.6] (nM)
b
WT
HC_S31G
HC_S31A
HC_S31L
c
WT
HC_Y32A
HC_Y100A
HC_Y100F
d
WT
HC_S103A
HC_S103L
e
WT
HC_N55A
HC_N55K
f
WT
HC_Y101A
HC_Y101F
g
WT
HC_G102L
HC_G102A
HC_R104A
h
WT
LC_C219A
i
WT
LC_H31A
LC_Y37A
LC_Y37F
j
WT
LC_N33A
LC_N33K
k
WT
LC_Y54F
l
WT
HC_Y54F
HC_Y54W
HC_Y54H
m
WT
HC_Y54A
HC_Y54G
HC_Y54L
HC_Y54V
HC_Y54M
n
WT
HC_Y54R
HC_Y54Q
HC_Y54D
o
WT + dsRNA
HC_Y54F + dsRNA
HC_Y54W + dsRNA
HC_Y54H + dsRNA
p
WT + dsRNA
HC_Y54R + dsRNA
HC_Y54Q + dsRNA
q
WT + dsRNA
HC_Y54A + dsRNA
HC_Y54L + dsRNA
HC_Y54V + dsRNA
HC_Y54M + dsRNA
r
Marker (kDa)
- + DTT
250
130
100
70
55
35
25
15
HC_WT
LC_C219A
SDS-PAGE

Supplementary Figure 3. Mutational analysis of nucleic acids binding by S9.6 variants. a comparison of Fab (black) and scFv (single-chain variable fragment, red) forms of S9.6 in hybrid binding. Values are mean ± s.d. n= 3 biologically independent samples. **(b-n)** Fluorescence polarization titrations of S9.6 variants binding to the 13-bp hybrid. Mutations are indicated in each panel. Values are mean ± s.d. n= 3 biologically independent samples. HC and LC indicate mutations in the heavy chain and light chain, respectively. **(o-q)** Fluorescence polarization titrations of 13-bp dsRNA with S9.6 variants. Values are mean ± s.d. n= 3 biologically independent samples. **r** SDS-PAGE analysis of LC_C219A variant in the presence or absence of reducing agents, showing the removal of the disulfide linkage. SDS-PAGE analysis was performed twice. Source data are provided as a Source Data file.

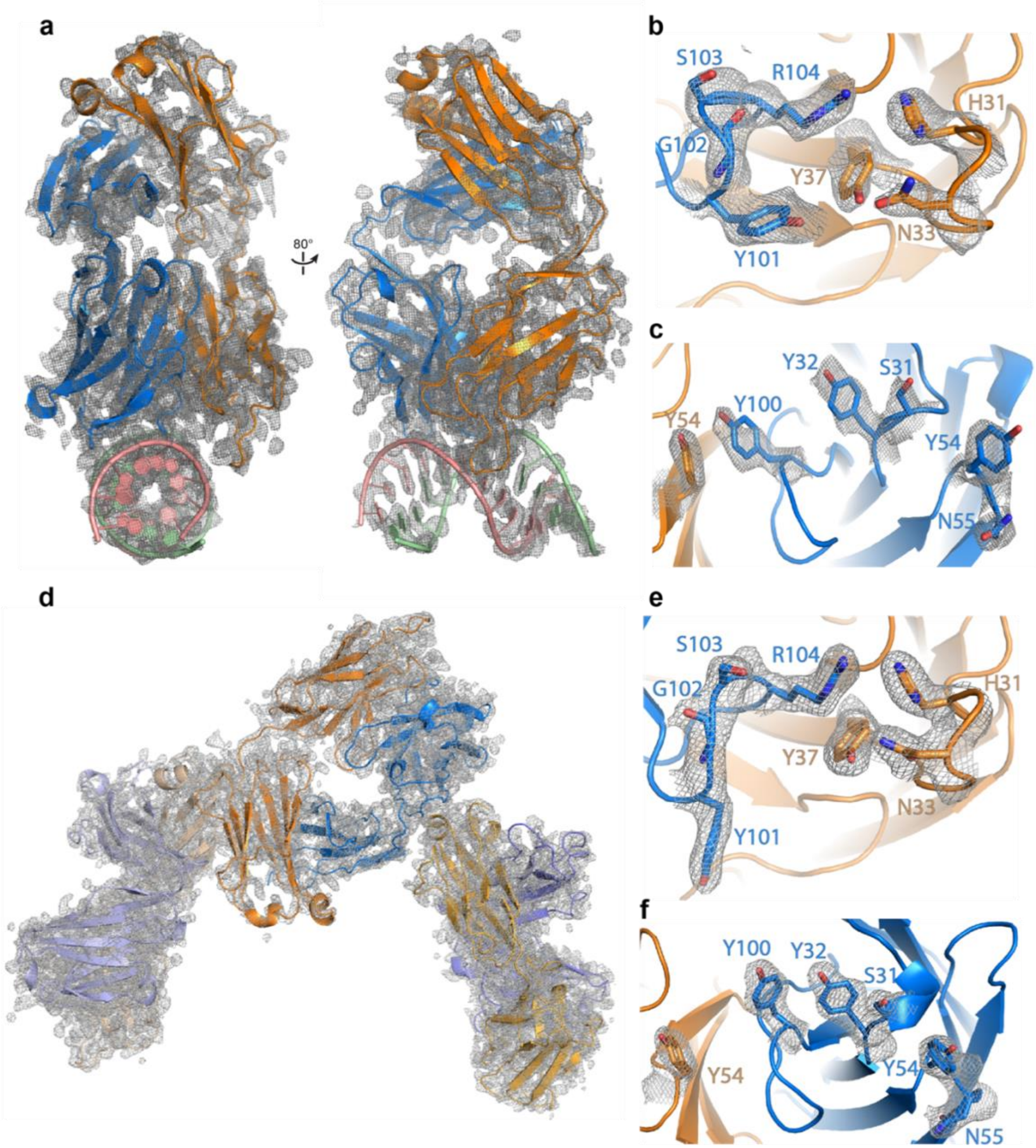

Supplementary Figure 4. Representative electron density. a Composite simulated anneal-omit 2Fo-Fc density calculated using the final model of S9.6 bound to a 13-bp DNA-RNA hybrid and superimposed with the final refined model, contoured at 1σ. **b** Portion of the map in **a** showing the S9.6 interface with the RNA strand. **c** Portion of the map in **a** showing the S9.6 interface with the DNA strand. **d** Composite simulated anneal-omit 2Fo-Fc density calculated using the final model of free S9.6 and superimposed with the final refined model, containing 3 molecules per asymmetric unit, contoured at 1σ. **e** Portion of the map in **d** showing residues involved in RNA strand recognition. **f** Portion of the map in **d** showing residues involved in DNA strand recognition. The heavy chain, the light chain, the RNA and DNA strand are shown in blue, orange, red and green, respectively.

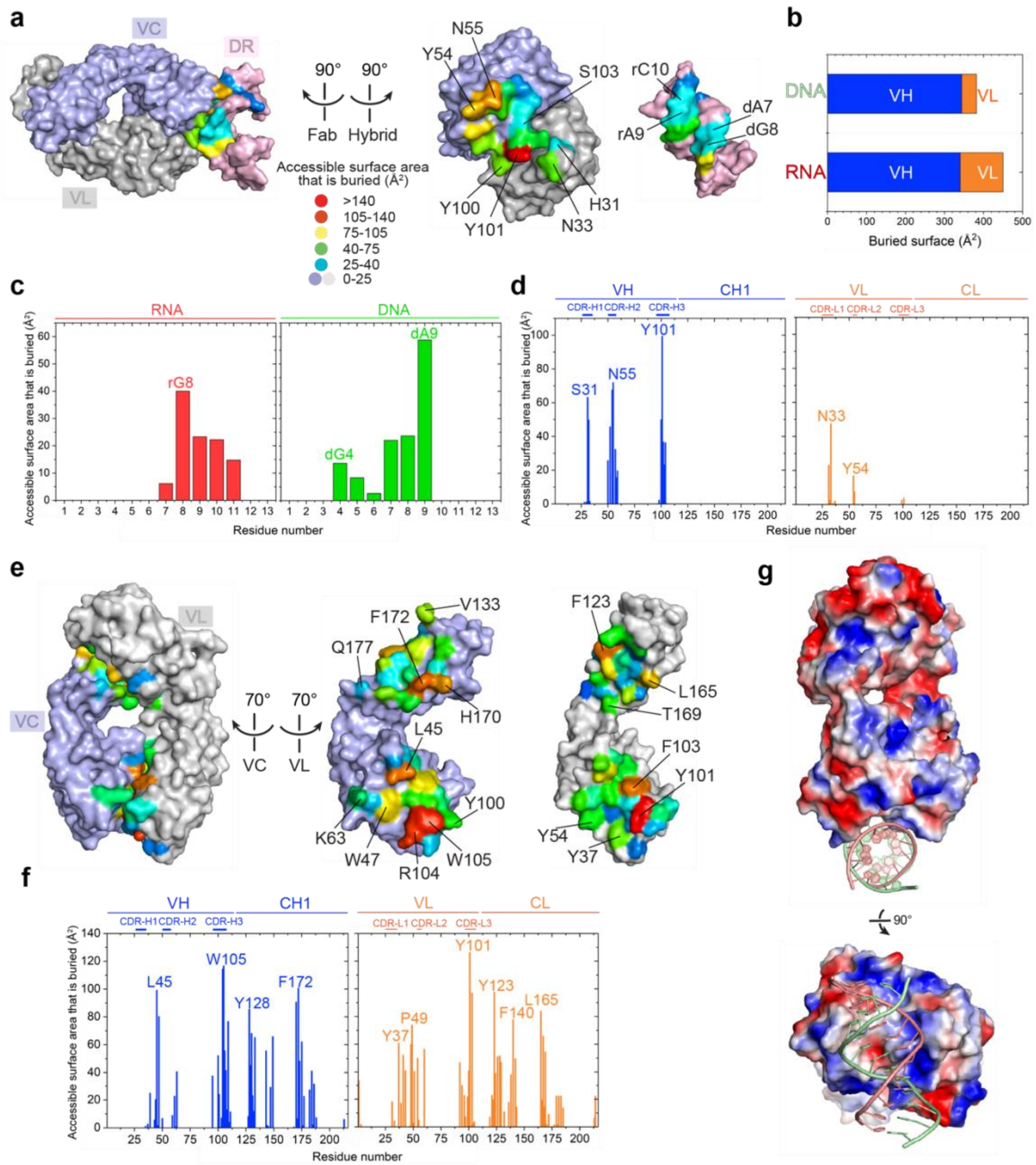

Supplementary Figure 5. Interfaces between S9.6 and hybrid and between HC and LC. a Open-book view of the S9.6 interface with the hybrid. Left: solvent-accessible surface colored according to area buried, from light blue or white (no burial) to red (>140 Å^2 per residue). Right: Outward-rotated views of the binding interface. **b** Plot of nucleic acid surface area buried by the heavy chain and light chain. **c** Plots of solvent-accessible surface area buried per residue on the RNA strand (red) and the DNA strand (green) by S9.6 binding. **d** Plots of solvent-accessible surface area buried per residue on S9.6 heavy chain (blue) and light chain (orange) by hybrid binding. **e** Open-book view of the S9.6 heavy chain interface with the light chain. Colored as in **a**. Right: Outward-rotated views of the binding interface. **f** Plots of solvent-accessible surface area buried per residue on the heavy chain (blue) and the light chain (orange) by Fab formation. **g** Electrostatic potential surface of S9.6.

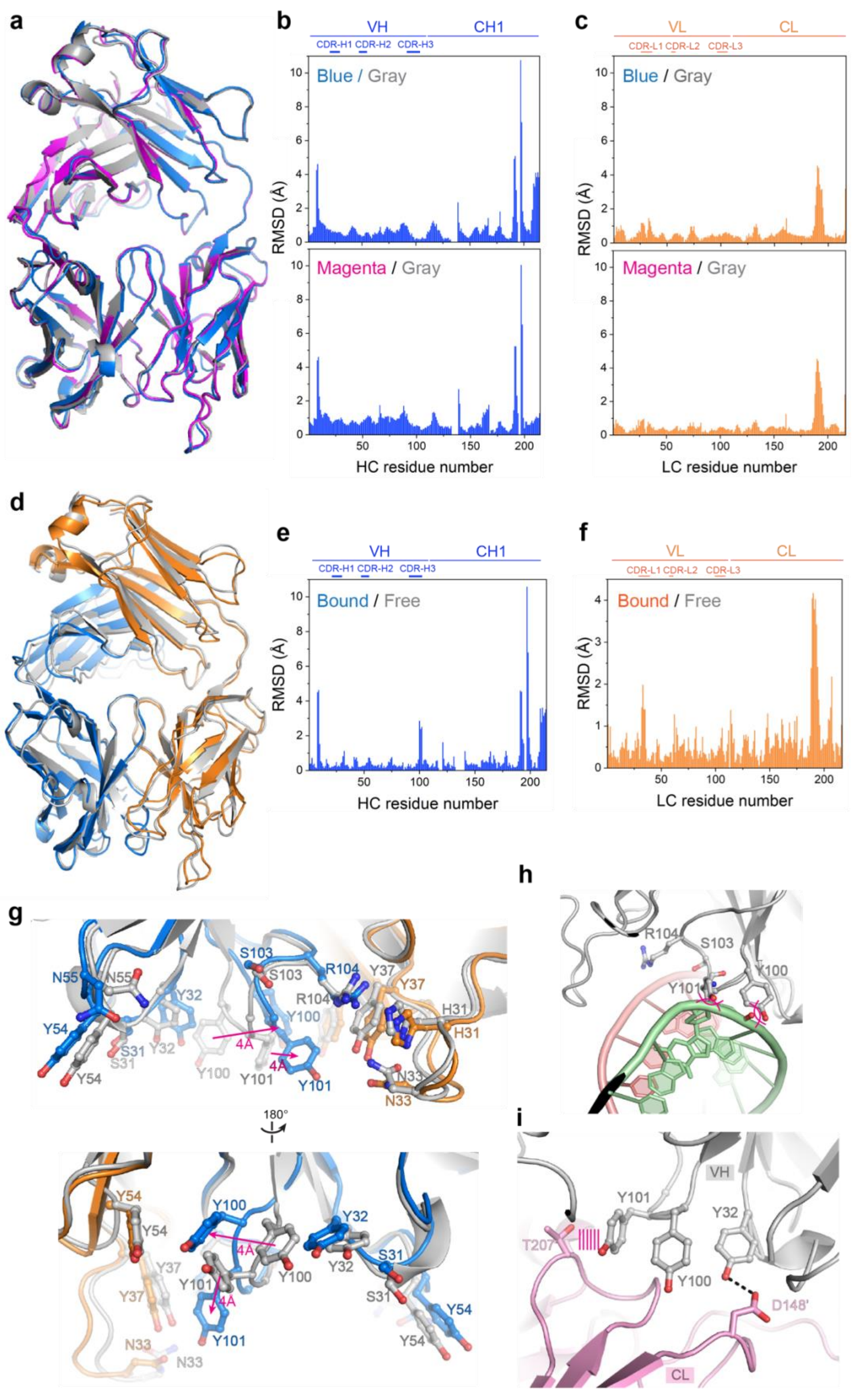

a
b
VH
CH1
CDR-H1 CDR-H2 CDR-H3
Blue / Gray
Magenta / Gray
RMSD (Å)
HC residue number
c
VL
CL
CDR-L1 CDR-L2 CDR-L3
Blue / Gray
Magenta / Gray
RMSD (Å)
LC residue number
d
e
VH
CH1
CDR-H1 CDR-H2 CDR-H3
Bound / Free
RMSD (Å)
HC residue number
f
VL
CL
CDR-L1 CDR-L2 CDR-L3
Bound / Free
RMSD (Å)
LC residue number
g
N55
N55
Y32
S103
S103
R104
R104
Y37
Y37
H31
H31
Y54
S31
S31
Y32
Y100
4Å
4Å
Y101
Y101
Y100
Y54
N33
N33
180°
Y54
Y54
Y100
Y32
Y37
Y37
Y101
Y100
Y32
S31
S31
Y54
Y101
N33
N33
Y54
h
R104
S103
Y100
Y101
i
VH
Y101
Y32
T207'
Y100
D148'
CL

Supplementary Figure 6. Structural comparison of S9.6 in free and hybrid-bound states. a Structural superposition of the three S9.6 molecules (in blue, magenta and gray) forming the asymmetric unit in the free S9.6 structure. **(b-c)** Per-residue root-mean-square deviations (RMSDs) between the three molecules shown in **a**. **d** Structural superposition of free S9.6 (gray) and S9.6 bound to hybrid (HC in blue; LC in orange). **(e-f)** Per-residue RMSDs between the free S9.6 and its hybrid-bound structure. For RMSD calculations, individual VH, VL, CH1 and CL domains were aligned to their counterparts. **g** Zoom-in view of **d** around the CDRs. Residues interacting with the hybrid are shown as sticks. Magenta arrows indicate the conformational changes in CDR-H3 upon hybrid binding. **h** Structural superposition of free S9.6 structure with the hybrid-bound structure near CDR H3 loop. Clashes are shown as overlapping magenta arcs. **i** Crystal packing contacts in free S9.6 structure involving CDR-H1 and H3 with a neighboring CL. Parallel lines indicate a potential stacking interaction.

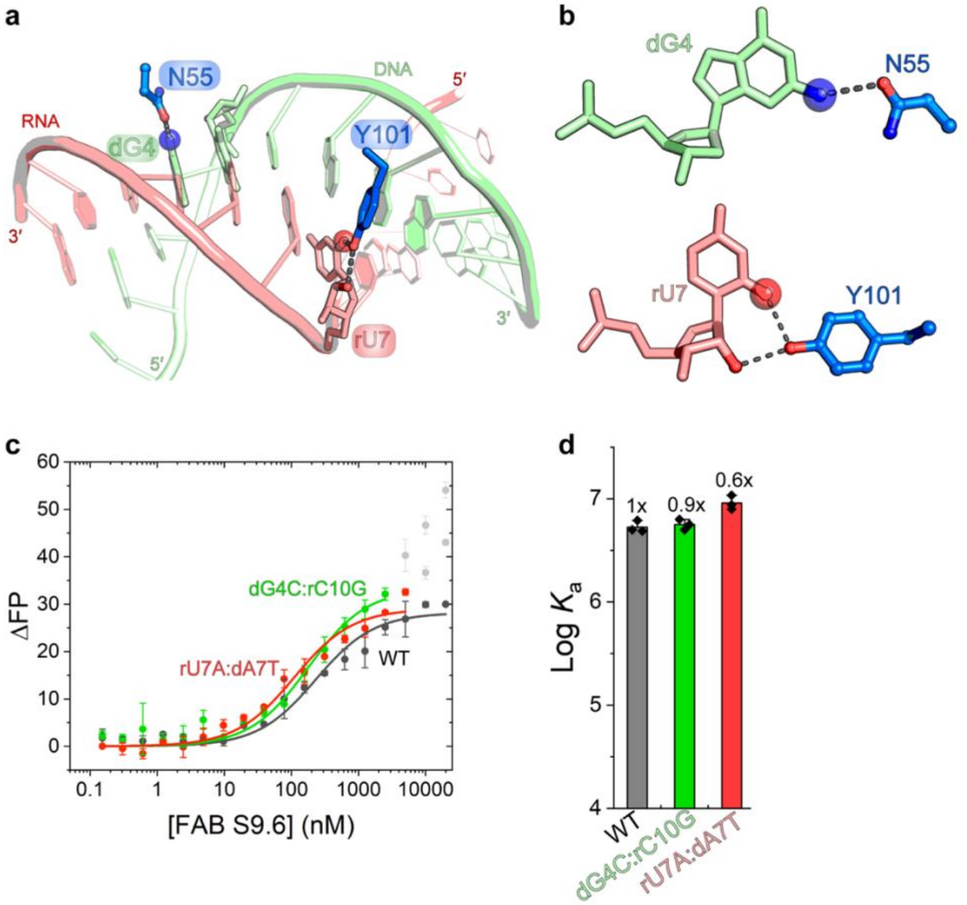

Supplementary Figure 7. Evaluation of nucleobase-specific contacts to S9.6. a Overall view of two nucleobase-specific contacts with S9.6. **b** Hydrogen-bonding patterns between N55 and Y101 of S9.6 Fab and dG4 and rU7 nucleobases of the hybrid, respectively. **c** Fluorescence polarization titrations using hybrids with swapped dG4C-rC10G and rU7A-dA7T pairs. Gray data points correspond to a non-specific, secondary binding event and were excluded from the fits for binding at the initial, high-affinity binding event. **d** Quantitation of data in **c** showing lack of effects of the base-pair swapping. Values are mean ± s.d. n= 3 biologically independent samples. Source data are provided as a Source Data file.

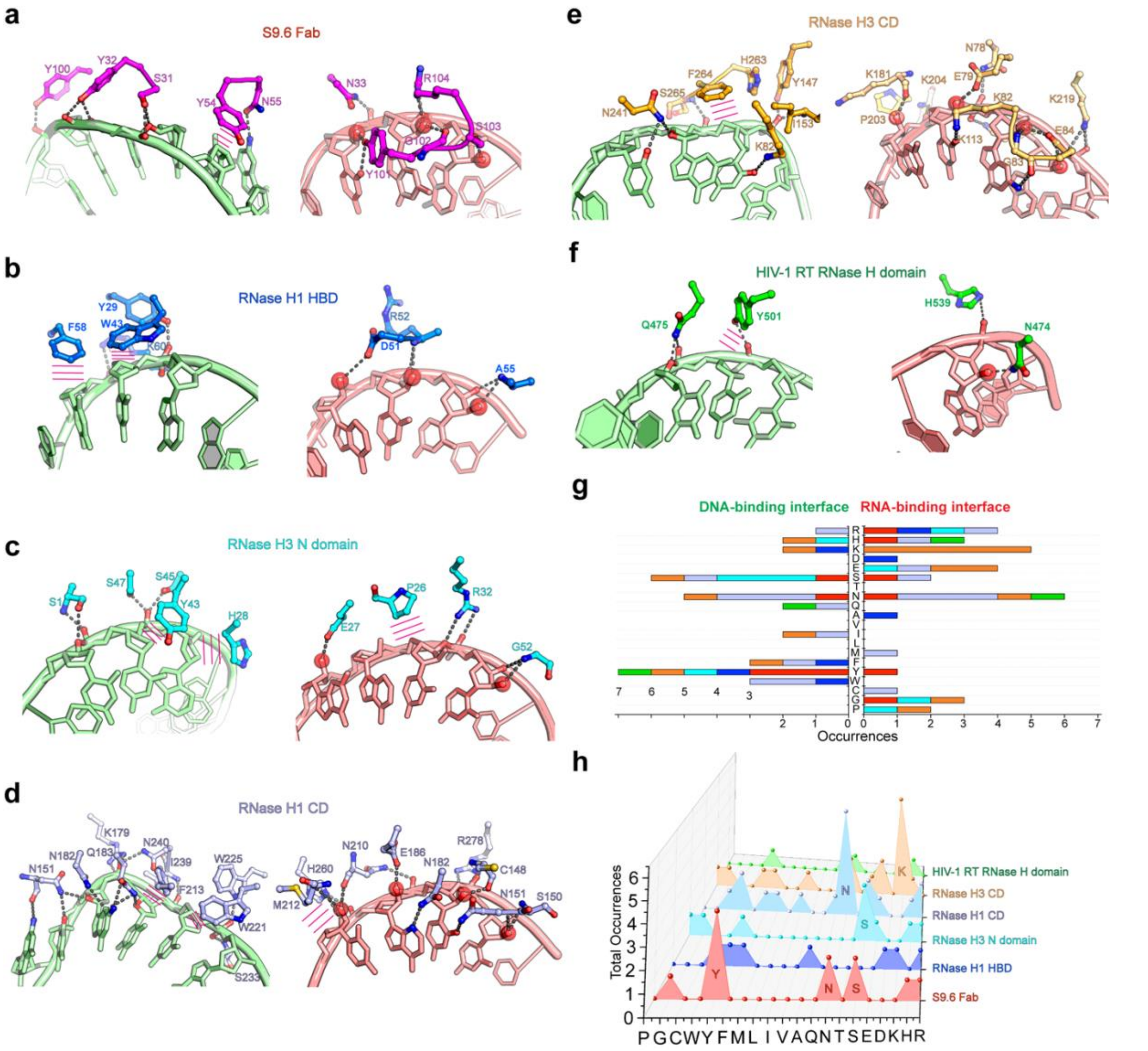

Supplementary Figure 8. Comparison of S9.6 and RNases H strategies for hybrid recognition. (a-f) DNA (left, green) and RNA (right, red) strand recognition by **a** S9.6 Fab (this work), **b** Human RNase H1 hybrid-binding domain (HBD, PDB: 3BSU[3]) **c** *Thermovibrio ammonificans* RNase H3 N-domain (PDB: 4PY5[4]), **d** RNase H1 catalytic domain (PDB: 2QK9[5]), **e** *T. ammonificans* RNase H3 catalytic domain (PDB: 4PY5[4]) and **f** RNase H domain of HIV-1 reverse transcriptase p66 (PDB: 6BSI[6]). **g** Occurrence of amino acids at the DNA or RNA binding interfaces of S9.6 Fab (red), RNase H1 HBD (blue), RNase H3 N-domain (cyan), RNase H1 catalytic domain (light blue), RNase H3 catalytic domain (orange) and RNase H domain of HIV-1 reverse transcriptase (green). **h** Sum of amino acid occurrences at the hybrid-binding interfaces in **g**.

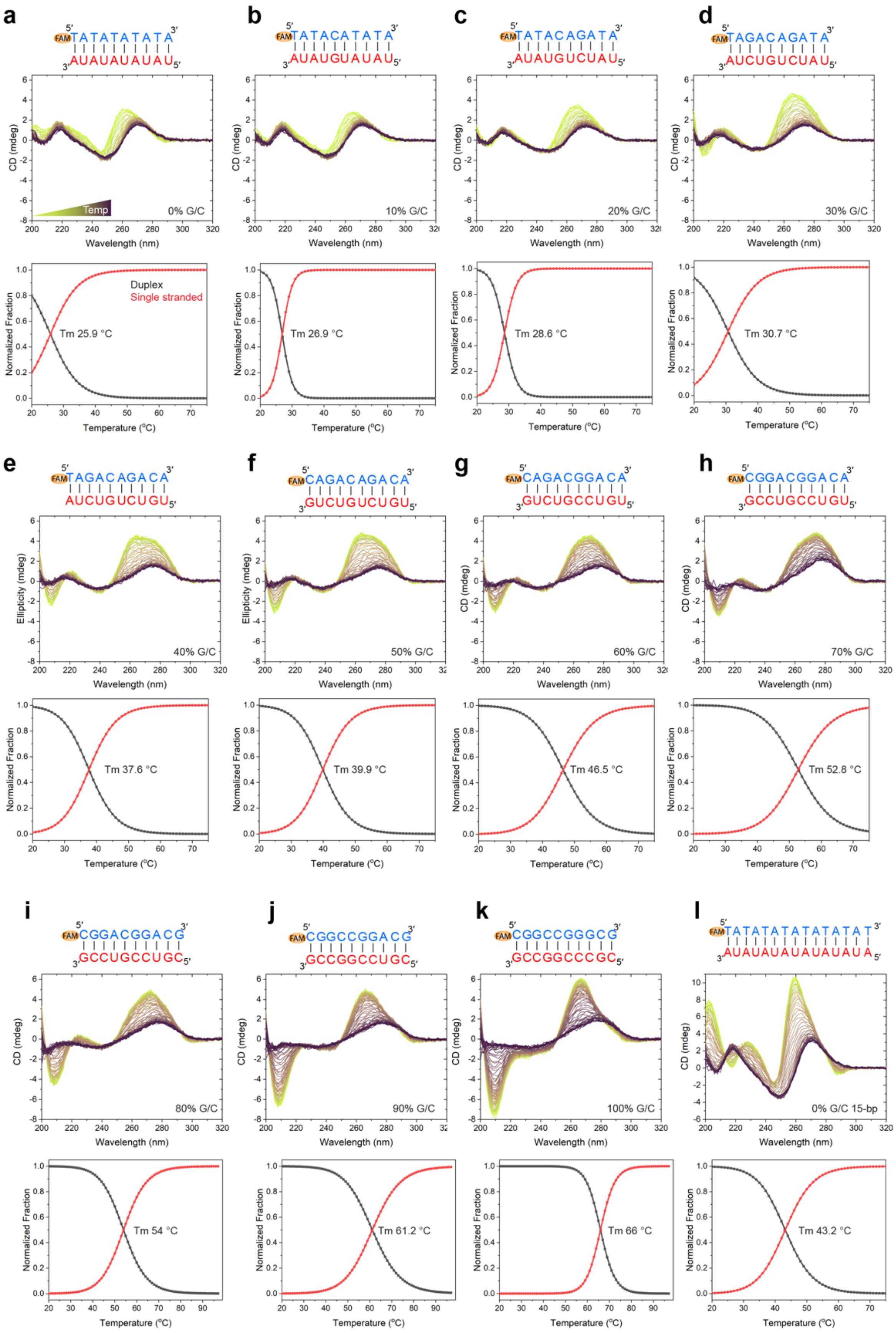
a
5′ FAM TATATATATA 3′
3′ AUAUAUAUAU 5′
CD (mdeg)
Temp
0% G/C
Wavelength (nm)
Normalized Fraction
Duplex
Single stranded
Tm 25.9 °C
Temperature (°C)
b
5′ FAM TATACATATA 3′
3′ AUAUGUAUAU 5′
10% G/C
Tm 26.9 °C
c
5′ FAM TATACAGATA 3′
3′ AUAUGUCUAU 5′
20% G/C
Tm 28.6 °C
d
5′ FAM TAGACAGATA 3′
3′ AUCUGUCUAU 5′
30% G/C
Tm 30.7 °C
e
5′ FAM TAGACAGACA 3′
AUCUGUCUGU 5′
Ellipticity (mdeg)
40% G/C
Tm 37.6 °C
f
5′ FAM CAGACAGACA 3′
3′ GUCUGUCUGU 5′
50% G/C
Tm 39.9 °C
g
5′ FAM CAGACGGACA 3′
3′ GUCUGCCUGU 5′
60% G/C
Tm 46.5 °C
h
5′ FAM CGGACGGACA 3′
3′ GCCUGCCUGU 5′
70% G/C
Tm 52.8 °C
i
5′ FAM CGGACGGACG 3′
3′ GCCUGCCUGC 5′
80% G/C
Tm 54 °C
j
5′ FAM CGGCCGGACG 3′
3′ GCCGGCCUGC 5′
90% G/C
Tm 61.2 °C
k
5′ FAM CGGCCGGGCG 3′
3′ GCCGGCCCGC 5′
100% G/C
Tm 66 °C
l
5′ FAM TATATATATATATAT 3′
3′ AUAUAUAUAUAUAUA 5′
0% G/C 15-bp
Tm 43.2 °C

Supplementary Figure 9. CD analyses of DNA-RNA hybrids with different GC contents. (a-k) Temperature-dependent circular dichroism (CD) measurements of 10-bp DNA-RNA hybrids with different GC contents. **l** Temperature-dependent CD measurement of a 15-bp DNA-RNA hybrid with 0% GC content. Sequences of hybrids used are shown on top of each panel with RNA and DNA nucleotides indicated in red and blue, respectively. The measured melting temperature (T_m) for each duplex is indicated.

References to Supplementary Figures:

1 Tan-Wong, S. M., Dhir, S. & Proudfoot, N. J. R-Loops Promote Antisense Transcription across the Mammalian Genome. *Mol Cell* **76**, 600-616 e606, doi:10.1016/j.molcel.2019.10.002 (2019).

2 Malig, M., Hartono, S. R., Giafaglione, J. M., Sanz, L. A. & Chedin, F. Ultra-deep Coverage Single-molecule R-loop Footprinting Reveals Principles of R-loop Formation. *J Mol Biol* **432**, 2271-2288, doi:10.1016/j.jmb.2020.02.014 (2020).

3 Nowotny, M. *et al.* Specific recognition of RNA/DNA hybrid and enhancement of human RNase H1 activity by HBD. *EMBO J* **27**, 1172-1181, doi:10.1038/emboj.2008.44 (2008).

4 Figiel, M. & Nowotny, M. Crystal structure of RNase H3-substrate complex reveals parallel evolution of RNA/DNA hybrid recognition. *Nucleic Acids Res* **42**, 9285-9294, doi:10.1093/nar/gku615 (2014).

5 Nowotny, M. *et al.* Structure of human RNase H1 complexed with an RNA/DNA hybrid: insight into HIV reverse transcription. *Mol Cell* **28**, 264-276, doi:10.1016/j.molcel.2007.08.015 (2007).

6 Tian, L., Kim, M. S., Li, H., Wang, J. & Yang, W. Structure of HIV-1 reverse transcriptase cleaving RNA in an RNA/DNA hybrid. *Proc Natl Acad Sci U S A* **115**, 507-512, doi:10.1073/pnas.1719746115 (2018).
