# Supplementary material for: Structural basis of R-loop recognition by the S9.6 monoclonal antibody
Source: Nat Commun. 2022 Mar 28;13:1641. doi: 10.1038/s41467-022-29187-7 (PMC8960830; doi:10.1038/s41467-022-29187-7)
Supplement: Supplementary file 3 — Description of Additional Supplementary Files [file 41467_2022_29187_MOESM3_ESM.pdf]

Description of Additional Supplementary Files

File Name: Supplementary Data 1
Description: Additional oligonucleotides used in this paper
