# Supplementary material for: Structural basis of R-loop recognition by the S9.6 monoclonal antibody
Source: Nat Commun. 2022 Mar 28;13:1641. doi: 10.1038/s41467-022-29187-7 (PMC8960830; doi:10.1038/s41467-022-29187-7)
Supplement: Supplementary file 5 — Reporting Summary [file 41467_2022_29187_MOESM5_ESM.pdf]

nature portfolio

Corresponding author(s): Jinwei Zhang

Last updated by author(s): Feb 25, 2022

Reporting Summary

Nature Portfolio wishes to improve the reproducibility of the work that we publish. This form provides structure for consistency and transparency in reporting. For further information on Nature Portfolio policies, see our Editorial Policies and the Editorial Policy Checklist.

Statistics

For all statistical analyses, confirm that the following items are present in the figure legend, table legend, main text, or Methods section.

n/a	Confirmed	
☐	☒	The exact sample size (*n*) for each experimental group/condition, given as a discrete number and unit of measurement
☐	☒	A statement on whether measurements were taken from distinct samples or whether the same sample was measured repeatedly
☒	☐	The statistical test(s) used AND whether they are one- or two-sided<br>*Only common tests should be described solely by name; describe more complex techniques in the Methods section.*
☒	☐	A description of all covariates tested
☒	☐	A description of any assumptions or corrections, such as tests of normality and adjustment for multiple comparisons
☒	☐	A full description of the statistical parameters including central tendency (e.g. means) or other basic estimates (e.g. regression coefficient) AND variation (e.g. standard deviation) or associated estimates of uncertainty (e.g. confidence intervals)
☒	☐	For null hypothesis testing, the test statistic (e.g. *F*, *t*, *r*) with confidence intervals, effect sizes, degrees of freedom and *P* value noted<br>*Give P values as exact values whenever suitable.*
☒	☐	For Bayesian analysis, information on the choice of priors and Markov chain Monte Carlo settings
☒	☐	For hierarchical and complex designs, identification of the appropriate level for tests and full reporting of outcomes
☒	☐	Estimates of effect sizes (e.g. Cohen's *d*, Pearson's *r*), indicating how they were calculated

Our web collection on statistics for biologists contains articles on many of the points above.

Software and code

Policy information about availability of computer code

Data collection	In-house data collection software was provided by SER-CAT beamline ID-22 at the Advanced Photon Source (APS) Argonne National Laboratory (ANL).
Data analysis	The X-ray diffraction data were indexed, integrated and scaled by XDS via Xia2 package. Molecular Replacement phasing was performed using Phaser. Model building was performed in Coot version 0.9. Refinement was performed using Phenix.Refine in PHENIX version 1.14. Binding measurements were plotted and analyzed in OriginPro Version 2018. CD measurements were analyzed using Global version 3.

For manuscripts utilizing custom algorithms or software that are central to the research but not yet described in published literature, software must be made available to editors and reviewers. We strongly encourage code deposition in a community repository (e.g. GitHub). See the Nature Portfolio guidelines for submitting code & software for further information.

Data

Policy information about availability of data

All manuscripts must include a data availability statement. This statement should provide the following information, where applicable:

- Accession codes, unique identifiers, or web links for publicly available datasets
- A description of any restrictions on data availability
- For clinical datasets or third party data, please ensure that the statement adheres to our policy

The atomic coordinates and structure factor amplitudes for the free S9.6 Fab and S9.6 Fab in complex with a 13-bp hybrid duplex have been deposited at the Protein Data Bank under accession codes 7TQA [https://www.rcsb.org/structure/7TQA] and 7TQB [https://www.rcsb.org/structure/7TQB]. All other data generated and analyzed during this study are provided in the Source Data file with this paper.

Field-specific reporting

Please select the one below that is the best fit for your research. If you are not sure, read the appropriate sections before making your selection.

☒ Life sciences ☐ Behavioural & social sciences ☐ Ecological, evolutionary & environmental sciences

For a reference copy of the document with all sections, see nature.com/documents/nr-reporting-summary-flat.pdf

Life sciences study design

All studies must disclose on these points even when the disclosure is negative.

Sample size	One crystal sample per dataset (due to full completeness). Biochemical and Biophysical assays were performed in independent biological triplicates in vitro.
Data exclusions	No data were excluded from the analysis.
Replication	All independent biological replications were successful and included.
Randomization	n/a this study did not use any organisms or participants.
Blinding	n/a this study did not use any organisms or participants.

Reporting for specific materials, systems and methods

We require information from authors about some types of materials, experimental systems and methods used in many studies. Here, indicate whether each material, system or method listed is relevant to your study. If you are not sure if a list item applies to your research, read the appropriate section before selecting a response.

Materials & experimental systems

n/a	Involved in the study	
☒	☐	Antibodies
☐	☒	Eukaryotic cell lines
☒	☐	Palaeontology and archaeology
☒	☐	Animals and other organisms
☒	☐	Human research participants
☒	☐	Clinical data
☒	☐	Dual use research of concern

Methods

n/a	Involved in the study	
☒	☐	ChIP-seq
☒	☐	Flow cytometry
☒	☐	MRI-based neuroimaging

Eukaryotic cell lines

Policy information about cell lines

Cell line source(s)	The Expi-CHO Expression System kit (Expi-CHO-s, ThermoFisher Scientific, Waltham, MA).
Authentication	Cell lines were purchased commercially and were not further validated.
Mycoplasma contamination	ExpiCHO-S (Thermo, A29127) have tested negative for mycoplasma contamination.
Commonly misidentified lines (See ICLAC register)	no commonly misidentified cell lines were used in the study
